# Relationship between Processing Method and the Glycemic Indices of Ten Sweet Potato (*Ipomoea batatas*) Cultivars Commonly Consumed in Jamaica

**DOI:** 10.1155/2011/584832

**Published:** 2011-10-29

**Authors:** Perceval S. Bahado-Singh, Cliff K. Riley, Andrew O. Wheatley, Henry I. C. Lowe

**Affiliations:** ^1^Department of Basic Meidcal Sciences, University of the West Indies, Mona Campus, Jamaica; ^2^Biotechnology Center, University of the West Indies, Mona Campus, Jamaica; ^3^Bio-Tech R&D Institute, Kingston, Jamaica; ^4^College of Health Sciences, University of Technology, Kingston, Jamaica

## Abstract

This study investigated the effect of different traditional cooking methods on glycemic index (GI) and glycemic response of ten Sweet potato (*Ipomoea batatas*) cultivars commonly eaten in Jamaica. Matured tubers were cooked by roasting, baking, frying, or boiling then immediately consumed by the ten nondiabetic test subjects (5 males and 5 females; mean age of 27 ± 2 years). The GI varied between 41 ± 5–93 ± 5 for the tubers studied. Samples prepared by boiling had the lowest GI (41 ± 5–50 ± 3), while those processed by baking (82 ± 3–94 ± 3) and roasting (79 ± 4–93 ± 2) had the highest GI values. The study indicates that the glycemic index of Jamaican sweet potatoes varies significantly with the method of preparation and to a lesser extent on intravarietal differences. Consumption of boiled sweet potatoes could minimize postprandial blood glucose spikes and therefore, may prove to be more efficacious in the management of type 2 diabetes mellitus.

## 1. Introduction

Sweet potatoes (*Ipomoea batatas*) are ranked as the seventh most commonly consumed carbohydrate-rich food source in the world [1] and one of the most important food crop in developing countries after rice, wheat, maize, and cassava [2]. It is a high yielding economic crop with over 90% of global production cultivated in developing countries. Compared to other crops, sweet potato is considered to be a superfood, with high nutritional value [3] and may be a better choice for consumption compared to potatoes (*Solanum tuberosum*). In the Caribbean, Jamaica is the leading producer of sweet potatoes. It is a major component of the Jamaican diet, where over 95% of the annual production (25,797,000 kg) is consumed locally as a source of digestible carbohydrate [4]. It is estimated that over 50% of the population (1.35 million) consume sweet potato at least once per week as part of their diet in a boiled, roasted, fried, or baked form. 

However, despite a dietary preference for sweet potatoes in Jamaica and other Caribbean countries, studies have indicated that complex carbohydrate-rich foods may have high glycemic indices resulting in potential harmful health effects [5–7] and the development of chronic diseases. Excess consumption of high glycemic index foods can lead to hyperinsulinemia, insulin resistance, weight gain, and possibly obesity, leading to insulin-resistant syndrome [8–10]. Recent studies have shown a positive correlation between the consumption of foods with high glycemic index and increased risk of chronic diseases such as type 2 diabetes, cardiovascular diseases, and cancer [11, 12]. 

Other studies have shown that not all complex carbohydrate-rich foods have high glycemic index [13, 14]. In contrast, foods with a low glycemic index can be beneficial in reducing the incidences of chronic diseases [13, 15]. Despite the name, sweet potato may be beneficial to persons with type 2 diabetes, resulting from the high fiber and manganese content, which could aid in stabilizing blood sugar levels and reduce insulin resistance. However, little information is available on the glycemic indices of sweet potatoes and their impact on blood glucose and glycemic response after consumption. 

As such, the present study was undertaken to investigate the effect of different processing methods on the GI and glycemic responses of ten sweet potato cultivars that are commonly eaten in Jamaica. 

## 2. Materials and Methods

### 2.1. Study Protocol and Subjects

The study was carried out using standard glycemic index testing protocol as outlined by Wolever et al. [16, 17]. Glucose was used as the reference food with a GI score of 100, tested in the subjects at baseline, midway, and at the end of the study. Subjects were appraised both verbally and in writing of the study protocol, and all gave written informed consent before participation. Ethics approval was granted by University Hospital of the West Indies Ethics Committee and conducted in accordance with its rules and regulations. Recruitment took place between February 2008 and March 2008 during which subjects were screened for any illness at the University of the West Indies Health Center. Anthropometric data and lifestyle factors were derived from questionnaires.

### 2.2. Inclusion/Exclusion Criteria

Only nondiabetic individuals between the ages of 25 and 45 years were eligible to participate in the study. Smokers, overweight, and obese individuals were excluded from the study. Emphasis was placed on subjects who were healthy, with an active lifestyle, without any diagnosed diseases, and not on prescribed medication. During the study, subjects were advised to continue their customary daily activities without any change in their physical activities.

### 2.3. Test Foods and Preparation

Freshly harvested, matured tubers from the ten most commonly eaten sweet potato cultivars (Dor, Quarter Million, Yellow Belly, Ganja, Watson, Clarendon, Minda, Ms Mac, Eustace, and Fire on Land) were collected from a local farm in St. Ann, Jamaica. 

The proximate compositions of the sweet potatoes were determined using the standard AOAC methods [18] and the available carbohydrate content calculated by difference [13, 19]. 

Samples used for the GI studies were thoroughly washed then cooked by boiling, roasting, baking, or frying on the day of glycemic index testing [14]. Foods processed by frying were peeled and cut into 50 grams wet weight available carbohydrate portions. They were then cut to 10 mm thickness (sweet potato wedges) and submerged in preheated, cholesterol-free vegetable cooking oil (Lider Brand, manufactured in Jamaica), until slightly brown. Foods processed by roasting were washed and cooked (skin intact) using preheated charcoal for 45 minutes in an open system. Foods processed by baking were washed and cooked (skin intact) in a preheated electric oven at 175°C for 45 minutes. Foods processed by boiling were washed, peeled, and cut into 25 mm slices. They were then cooked in water (gentle boiling) with the lid of the cooking vessel on for 20 minutes, followed by simmering heat (lid of cooking vessel off) for a further 10 minutes. After the boiling process, the available carbohydrate content was determined, to assess the loss of sugars that may have occurred during cooking. The foods were then cut into 50 grams available carbohydrate portions, required for GI analysis.

### 2.4. Glycemic Index Experimental Design

A randomized cross-over study design was conducted with 10 healthy nondiabetic subjects (5 males and 5 females). Fifty grams (50 g) available carbohydrate portions of the test foods were administered to the subjects on separate mornings after a 10–12-hour overnight fast. For individual subjects the tests were given 4 days apart [13]. Subjects were asked not to perform any strenuous activities, take long walks, or consume alcohol on the day of glycemic index determination. They were asked to remain seated for the duration of the test. Test meals were consumed within 10 minutes and supplemented with 250 mL of water. Capillary pricked-finger blood samples were taken (3-4 drops) at baseline (0 mins), 15, 30, 45, 60, 90, and 120 minutes after the meal was consumed. Blood samples were collected into heparin tubes and stored at −20°C before glucose analysis. Blood glucose was determined using the glucose oxidase method using a UV/Visible Ultraspec spectrophotometer (Model 1100 pro).

The incremental areas under the curve (IAUC), excluding the area beneath the fasting level, was calculated geometrically [16]. The GI was then calculated by expressing the glycemic response area for the sweet potato as a percentage of the mean response area of the reference food (glucose) taken by the same subjects [16].

### 2.5. Statistical Power and Statistical Analysis

The power of the tests with 10 subjects was expected to have 80% power to detect differences in glycemic response of about 20% in the incremental areas under the glucose response curves (IAUCs) above the fasting level between the foods. This calculation assumed a variation of 22% within subjects [16]. Statistical analyses were performed using the Statistical Package for Social Sciences version 12.0 (SPSS Inc., Chicago, Ill). The changes in blood glucose levels after consumption of the different sweet potato varieties by time interval were analyzed by repeated-measurement analysis of variance (ANOVA) and Duncan's multiple range tests. Differences in means were considered statistically significant at *P* < 0.05. 

## 3. Results

The 10 subjects (5 males and 5 females), all of African descent, were between ages 25 and 45 years with a mean age of 27 ± 2 years and BMI ranging from 22.91 kg/m^2^ to 28.32 kg/m^2^ (24.65 ± 0.4 kg/m^2^). The carbohydrate content of the unprocessed sweet potato tubers ranged from 26.86 [g/100 g] to 31.74 [g/100 g] with Fire on Land having the lowest and Minda the highest content. Dietary fiber content of the ten varieties also differed significantly ranging from 2.87 ± 0.03 [g/100 g] to 3.74 ± 0.04 [g/100 g] (Table 1). Actual serving sizes containing 50 g available carbohydrate [g/100 g] for the test meals were larger for boiled foods than those roasted, baked, or fried (Table 2). 

**Figure 1:**
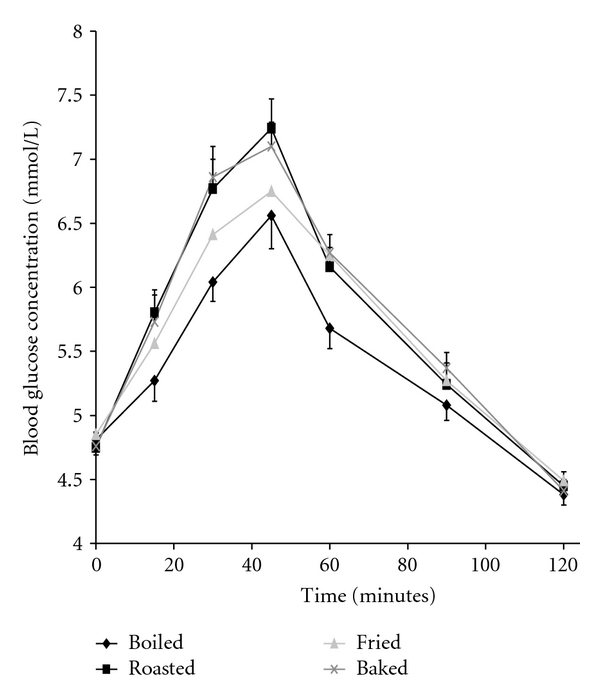
Mean glycemic responses elicited by 50 g available carbohydrate portions of all ten sweet potato cultivars processed by boiling (solid diamond), baking (x), roasting (solid square), and frying (gray triangle).

Table 3 shows the glycemic index values of the different sweet potato varieties calculated relative to the reference food (glucose GI = 100) and classified as high (70 to 100), intermediate (55 to 69), or low (<55). The GI values were significantly lower for foods processed by boiling (Ganja variety having the lowest GI = 41 ± 5) when compared to the other processing methods (*P* < 0.05). Foods baked and roasted had high GI values, while those fried (sweet potato wedges) had intermediate to moderately high GI values (63 ± 2 to 77 ± 4). Table 4 shows the incremental area under the glucose response curve for the sweet potato varieties studied, while Figure 1 shows the mean glycemic responses of all the sweet potato cultivars processed by the different cooking methods.

## 4. Discussion

The classification of foods based on their glycemic index has dispelled the repeatedly suggested dietary notion that carbohydrate-rich foods have deleterious health effects, and, as such, consumption should be limited [20, 21]. In fact, there are numerous evidence-based studies which dismiss the negative view of carbohydrate-rich foods and clearly demonstrate that “not all carbohydrates are created equal” [13, 14, 22]. Furthermore, variations in the physiochemical properties of complex carbohydrates have been shown to elicit dissimilar physiological effects when consumed [23]. Also, some complex carbohydrate-rich foods are undeniably beneficial and do not cause blood glucose levels to spike any greater than some simple sugars. However, food preparation is important and should be considered, as the method of cooking can alter the structure and nature of the starches resulting in significant effects on postprandial blood glucose responses [24]. 

The results from this study show that the processing of sweet potatoes by boiling illicits lower GI values when compared to frying, baking, and roasting (Table 3). This may be linked to the chemical structure of starches, that is, the amylose-amylopectin ratio [25]. Miller et al. [13] and Goddard et al. [26] reported that rice with higher amylose content was accompanied by lowered metabolic response and lower GI values. Boiling is believed to induce gelatinization, thereby permanently disrupting the amylose-amylopectin structure of the starch complex, thus making it more readily accessible by digestive enzymes. At the same time retrograded amylose is indigestible due to the presence of stronger hydrogen bonding in comparison with retrograded amylopectin [27]. Concomitantly, greater amounts of resistant starches (RS1, RS2, and RS3) may have been retained in the boiled foods.

Furthermore, as these foods cool, the possibility of forming R3-resistant starches (retrograded starches) increases. This occurs as the starches undergo recrystallization due to the formation of intermolecular hydrogen bonds. Other resistant starches (R1 and R2) present in the foods after the leaching of free sugars during the boiling process also play a role in retarding the enzymatic degradation of the starches, thus reducing the glycemic response. In a similar study, Englyst and Cummings [24] reported that about 7% of starch in reheated boiled potatoes (*Solanum tuberosum *sp.) escapes digestion in the ileum compared with about 3% in freshly cooked potato. 

Sweet potato is often eaten fried “termed sweet potato wedges” and is a popular alternative to French fries (Irish Potatoes—*Solanum tuberosum*). The results indicate that fried sweet potatoes had intermediate to moderately high GI (Table 4). The GI of Ms Mac variety was similar to that of French fries (*Solanum tuberosum *sp.) reported by Fernandes et al. [28]. The lower glycemic indices observed on frying compared to baking and roasting could be attributed to the increased fat content resulting in retardation in starch degradation, consequently delaying gastric emptying and glycemic response. This principle was supported by Fernandes et al. [28] who reported similar GI values for French fries from *Solanum *sp. In addition, *in vitro *studies [29] revealed that the frying process increases the amount of RS in potatoes. This decreases the rate of hydrolysis of the amylose-amylopectin starch structure resulting in a lowered glycemic response [30]. In addition, studies by Holm et al. [31] and Leeman et al. [30] suggested that amylose is prone to react with lipids to form amylose-lipid complexes thus reducing the rate of amylolysis and resulting in lower glycemic responses and GI values. 

The study also shows that cooking by roasting and baking resulted in spikes in postprandial blood glucose levels for all the sweet potato varieties studied (81–94) as seen in the IAUC in Table 4. Cooking with the skin intact increases the availability of free sugars which are immediately hydrolyzed by salivary amylase and are instantly absorbed. Additionally the absence of a water-rich environment as similar to that of boiling would have resulted in less starch gelatinization and by extension production of lower levels of retrograded/RS 3 starch. 

Additionally, the GI values of these sweet potato cultivars studied could be substantiated by correlating the texture of the sweet potato cultivars as described by studies done by Henry et al. [32] who reported a strong positive correlation between GI and the texture rating in commercially available potatoes eaten in Great Britain. Furthermore, intravarietal variations could be related to differences in the starch physicochemical properties and maturity index of the different varieties used. Additionally, it has been reported that precooking or allowing the food to cool and then reheating before consumption may elicit a lower glycemic response compared with consumption immediately after cooking [28, 33]. As such, further studies can be carried out to investigate the effects of starch properties, maturity index, precooking, cooling, and reheating on these sweet potatoe cultivars on GI. 

Since sweet potato is a major staple in the Jamaican and the wider Caribbean diet, the results suggest that health conscious individuals and persons with diabetes should consider avoiding the consumption of baked or roasted sweet potatoes and should not be misguided by the confounding fact that it is a highly nutritious root vegetable. Similarly, the identification of cooking methods of sweet potatoes with lower glycemic responses may help reduce the GI and glycemic load of the Jamaican diet which could prove beneficial in the management and prevention of other chronic diseases. 

## 5. Conclusion

This study is the first to report variations in the glycemic indices and blood glucose responses among the sweet potato (*Ipomoea batatas*) cultivars eaten in Jamaica and the wider Caribbean with respect to different cooking methods. Tubers processed by boiling had the lowest glycemic, index while those roasted and baked had significantly higher glycemic indices. Generally Ganja had the overall lowest glycemic index and Eustace the highest among the sweet potato varieties studied. 

The results therefore indicate that method of food preparation significantly impacts on the glycemic index of Jamaican sweet potatoes (*P* < 0.005). Consumption of boiled sweet potatoes may minimize the risk of postprandial blood glucose spikes, thereby reducing diabetic and cardiovascular disease indices and thus may prove to be more efficacious in the management of type 2 diabetes mellitus. Increased amount of hydrogenated fats in the diet is potentially unhealthy; hence, it is advisable to limit the consumption of fried foods [14]. 

## Figures and Tables

**Table 1:** Proximate composition of 10 unprocessed Jamaican sweet potato (*Ipomoea batatas*) cultivars (100 g).

Sweet potato varieties	Moisture content (g)		Protein content [N × 6.25] (g)		Fat content (g)		Fiber Content (g/100 g)		Total sugars (g)		Ash content (g)		Available Carbohydrate content (by difference) (g/100 g)
	Mean	SE	Mean	SE	Mean	SE	Mean	SE	Mean	SE	Mean	SE	
Dor	65.91	1.39	0.97	0.04	0.25	0.01	3.08	0.06	3.75	0.14	0.96	0.03	28.83
Quarter Million	67.06	0.52	1.54	0.04	0.27	0.01	2.89	0.03	4.89	0.29	1.02	0.03	27.22
Yellow Belly	65.11	1.28	0.81	0.05	0.28	0.01	3.53	0.02	3.62	0.22	1.04	0.02	29.23
Ganja	63.27	0.65	1.62	0.18	0.31	0.02	2.97	0.03	3.92	0.13	1.05	0.03	30.78
Watson	64.96	1.19	1.55	0.06	0.32	0.02	3.46	0.05	4.21	0.25	0.91	0.02	28.80
Clarendon	62.54	1.26	1.53	0.05	0.29	0.02	3.74	0.04	4.17	0.34	1.18	0.05	30.72
Minda	62.35	0.78	1.29	0.08	0.23	0.01	2.99	0.05	4.86	0.25	1.40	0.02	31.74
Ms Mac	65.73	0.26	1.20	0.04	0.24	0.01	2.87	0.03	4.85	0.33	0.99	0.02	28.97
Eustace	67.21	2.24	0.58	0.03	0.33	0.02	3.52	0.04	5.01	0.54	1.01	0.03	27.35
Fire on Land	67.79	1.32	1.02	0.05	0.35	0.02	3.04	0.03	3.26	0.13	0.94	0.03	26.86

Values are means ± SEM. *n* = 4.

**Table 2:** Available carbohydrate (CHO g) in 100 g unprocessed sweet potato cultivars* and serving sizes^§^ used for glycemic index determination.

Sweet potato varieties	Food processing methods			
	Boiled		Roasted, baked, and fried	
	Available CHO (g/100 g)	Serving size (g)	Available CHO (g/100 g)	Serving size
Dor	21.22	235	28.83	173
Quarter Million	19.78	252	27.22	183
Yellow Belly	20.32	346	29.23	171
Ganja	21.52	232	30.78	162
Watson	18.98	263	28.80	173
Clarendon	22.28	218	30.72	162
Minda	21.43	233	31.74	157
Ms Mac	19.81	252	28.97	172
Eustace	18.32	272	27.35	182
Fire on Land	20.45	244	26.86	186

*Except for 100 grams of boiled sweet potatoes.

^§^Containing 50 g available carbohydrate.

**Table 3:** Glycemic indices^§^ of selected Jamaican sweet potato (*Ipomoea batatas*) varieties determined by different cooking methods.

Sweet potato varieties	Glycemic index							
	Boiled		Fried		Baked		Roasted	
	Mean	SE	Mean	SE	Mean	SE	Mean	SE
Dor	47^a^	3	76^b^	4	83^c^	6	86^c^	4
Quarter Million	49^a^	4	70^b^	6	94^c^	3	91^c^	2
Yellow Belly	50^a^	3	72^b^	4	86^c^	2	85^c^	2
Ganja	41^a^	5	69^b^	3	82^c^	3	79^c^	4
Watson	43^a^	4	67^b^	4	85^c^	2	87^c^	2
Clarendon	46^a^	5	73^b^	3	83^c^	3	81^c^	4
Minda	49^a^	4	68^b^	3	91^c^	3	89^c^	3
Ms Mac	45^a^	3	63^b^	2	87^c^	4	85^c^	4
Eustace	49^a^	5	77^b^	4	93^c^	5	93^c^	2
Fire on Land	46^a^	4	75^b^	3	87^c^	4	90^c^	3

Superscripts in rows sharing different letters are significantly different (*P* < 0.05).

Values are means ± SEM for *n* = 10 subjects.

^§^Glycemic index for each sample was calculated by expressing the IAUC as a percentage of the mean response area of glucose as outlined by Wolever et al. [16].

**Table 4:** Incremental areas under the glucose response curves for ten sweet potato (*Ipomoea batatas*) cultivars processed by different cooking methods and glucose standard.

Sweet potato varieties	Incremental area under glucose response curve				
	Boiled	Fried	Baked	Roasted	Glucose standard
Dor	51 ± 13^a^	120 ± 21^b^	156 ± 43^c^	134± 32^d^	179 ± 53^e^
Quarter Million	47 ± 19^a^	132 ± 25^b^	188 ± 39^c^	192 ± 52^c^	183 ± 41^c^
Yellow Belly	65 ± 15^a^	144 ± 32^b^	174 ± 25^c^	166 ± 33^c^	169 ± 32^c^
Ganja	46 ± 17^a^	129 ± 23^b^	169 ± 15^c^	132 ± 14^b^	175 ± 53^d^
Watson	44 ± 18^a^	118 ± 19^b^	173 ± 27^c^	164 ± 31^d^	159 ± 33^e^
Clarendon	49 ± 23^a^	142 ± 36^b^	176 ± 20^c^	142 ± 57^b^	165 ± 42^d^
Minda	54 ± 22^a^	131 ± 25^b^	184 ± 43^c^	189 ± 45^c^	174 ± 51^d^
Ms Mac	55 ± 13^a^	126 ± 19^b^	171 ± 53^c^	174 ± 43^c^	184 ± 64^d^
Eustace	64 ± 29^a^	142 ± 36^b^	187 ± 54^c^	201 ± 66^d^	178 ± 43^c^
Fire on Land	43 ± 16^a^	122 ± 16^b^	185 ± 81^c^	187 ± 53^c^	164 ± 47^d^

Superscripts in rows sharing different letters are significantly different (*P* < 0.05).

Values are means ± SEM for *n* = 10 subjects.
